# Effects of unilateral, bilateral, and combined unilateral+bilateral complex resistance training on bench press and squat strength in adolescent boxers

**DOI:** 10.3389/fphys.2024.1321519

**Published:** 2024-03-04

**Authors:** Yang Liu, Xiuxia Liu, Jiaxian Geng

**Affiliations:** ^1^ Sports Science Postdoctoral Mobility Station, Chengdu Sport University, Chengdu, China; ^2^ School of Physical Education, Nanchang Normal University, Nanchang, Jiangxi, China; ^3^ Department of Physical Education, Xiamen University, Xiamen, China; ^4^ Institute of Physical Education, Huzhou University, Huzhou, Zhejiang, China

**Keywords:** adolescent boxers, resistance training, unilateral training, bilateral training, strength exercises

## Abstract

**Objective:** To assess the effects of 8 weeks of unilateral (UNI), bilateral (BI), and combined unilateral + bilateral (UNI + BI) resistance training on bench press and squat strength in adolescent boxers.

**Methods:** Using the Gym Aware linear accelerometer, free-weight squat and bench press strength exercises were evaluated after an 8-week training intervention. Thirty adolescent boxers were randomly assigned to three groups: UNI, height: 1.73 ± 0.08 m, weight: 55.42 ± 5.85 kg; UNI + BI, height: 1.7 ± 0.06 m, weight: 54.73 ± 5.33 kg; and BI, height: 1.74 ± 0.06 m, weight: 59.67 ± 8.39 kg. Each group followed their designated UNI/BI/UNI + BI compound resistance training protocols, and the effects of 8 weeks of single-sided and bilateral intervention training on the performance of free-weight squat and bench press exercises at 30%, 50%, and 80% of 1-repetition maximum (1RM) were evaluated.

**Results:** Significant improvements were observed in the 30% 1RM, 50% 1RM, and 80% 1RM outcomes for both squat and bench press exercises before and after the interventions (*p* < 0.05, *p* < 0.01). In the intergroup comparison, GymAware measurements revealed that the UNI and UNI + BI groups exhibited superior peak power values for squat and bench press exercises at 30% 1RM compared to the BI group.

**Discussion:** UNI and UNI + BI training led to significantly higher output power values in bench press and squat exercises at 30% 1RM compared to the BI training group.

## 1 Introduction

Complex training (CT), also known as complex resistance training, is a methodology that concurrently enhances muscle strength and explosive power within a single training session or unit. CT refers to the integration of high-load resistance training with subsequent rapid eccentric-concentric exercises, known as plyometrics, which mimic the biomechanics of the preceding resistance training. This approach capitalizes on the post-activation potentiation (PAP) effect induced by heavy resistance training, resulting in improved efficiency during subsequent explosive exercises. CT represents a fusion of traditional heavy resistance training and rapid eccentric-concentric training, offering a comprehensive training strategy to optimize strength and explosive power.

Research into CT has centered on its effectiveness in enhancing strength and explosive power. In nearly all contact sports, athletes are required to possess a combination of strength and explosive power, as indicated by performance assessment metrics including vertical jumping (Pagaduan and Pojskic, 2020), sprinting, and peak power of the upper and lower limbs (Gourgoulis et al., 2003; Alves et al., 2010). Disciplines such as soccer, basketball, volleyball, track and field, as well as winter sports, involve this multifaceted performance evaluation.

Pagaduan and Pojskic (2020) conducted a comprehensive literature review investigating the impact of CT on vertical jump performance, and revealed that CT led to a significantly greater enhancement in vertical jump performance compared to standalone plyometric training (Z = 4.15, *p* = 0.01). Specifically, CT resulted in a substantial 15.9% increase in vertical jump performance (95% CI: 2.71–4.66 cm). Moreover, when contrasted with a control group that performed plyometric exercises, CT showcased a marked improvement in vertical jump scores, displaying an 8.8% enhancement (95% CI: 1.48–2.06 cm). These results show that CT amalgamates the benefits of two distinct training types, thereby yielding superior performance enhancement compared to resistance training and standalone plyometric training.

The synthesis of these studies underscores the efficacy of CT in concurrently augmenting strength and explosive power. We aimed to assess the effects of 8 weeks of unilateral, bilateral, and combined unilateral + bilateral resistance training on bench press and squat strength in adolescent boxers, to establish CT as a promising approach for athletes in diverse sporting disciplines who aim to optimize their performance.

## 2 Materials and methods

### 2.1 Participants

Male lightweight boxers were selected from the Jiangxi Province and Nanchang City boxing teams. Inclusion criteria for the experimental participants were as follows: 1) adolescent athletes aged between 16 and 18 years; 2) athletes with a sports classification of at least second level or with training experience of at least 4 years; 3) participants categorized as lightweight, based on weight classification; 4) orthodox stance with the dominant side as the rear hand side; and 5) possession of a minimum of 1 year of foundational strength training experience, including techniques in weightlifting, bench press, or squat exercises. Athletes meeting these criteria were encoded as S1 to S36 and grouped into unilateral (UNI), bilateral (BI), and unilateral + bilateral (UNI + BI) groups using a random number table. The start point was confirmed using a stopwatch, ensuring alignment with the *x* and *y*-axes, and the last two digits of the subsequent three data points were extracted for numerical consistency. The group distribution was balanced, with two adjustments made midway, and six participants were excluded due to various reasons such as participating in competitions or sustaining injuries. Data from 30 participants were included for analysis, with each group (UNI, BI, and UNI + BI) comprising 10 individuals. Morphological indicators within the three groups were assessed using the KS test and Brown–Forsythe test for homogeneity of variance, all yielding *p*-values >0.05, thereby confirming the suitability of the overall distribution. To ensure precise control of experimental conditions, it was essential to ascertain that there were no significant differences in baseline indicators (such as height, body mass, etc.) among the three groups.

### 2.2 Instrumentation and equipment

A linear acceleration device equipped with the GymAware (Australia:a linear positional transducer) (Harris et al., 2021) was utilized to assess changes in upper and lower limb power output of the athletes before and after the intervention. The GymAware system utilizes a linear position sensor that can be attached to a barbell or suspended resistance training equipment. It calculates displacement and time to derive velocity and average velocity, enabling the calculation of peak power and peak velocity. GymAware stands as a gold standard instrument for the evaluation of upper and lower limb athletic performance (Harris et al., 2010). The system (Grgic et al., 2020), rooted in velocity-based training standards, was employed as the benchmark to monitor and evaluate upper and lower limb athletic performance. Previous literature validates the high reliability and validity of linear sensors in physical fitness monitoring (Orange S. et al., 2018), ensuring precise monitoring during training.

### 2.3 One-repetition maximum (1RM) testing

Prior to commencing the training regimen, the GymAware system was employed to assess the 1RM strength qualities of the athletes in squat and bench press exercises. Subsequent training was then tailored based on the varying loads corresponding to different percentages of 1RM. We included testing at maximal repetitions of 30%, 50%, and 80% of 1RM.

The training design of the complex resistance training apparatus was standardized at 85% 1RM combined with rapid contraction training, corresponding to velocity ranges of <0.5 m/s + 1.3 m/s - 1 m/s or >1.3 m/s. The velocity range of 1.3–1 m/s represented near maximal effort (squatting with near-vertical jump and barbell elevation during bench press), 0.9–0.5 m/s targeted power (explosive force), and <0.5 m/s aimed at absolute strength training. GymAware software was employed for 1RM prediction and to generate force-velocity curves. Absolute strength indices were categorized using GymAware grouping, wherein athletes were organized into groups for monitoring purposes. Barbell velocity for each load was monitored, facilitating fatigue monitoring and ensuring scientifically grounded training.

**FIGURE 1:**
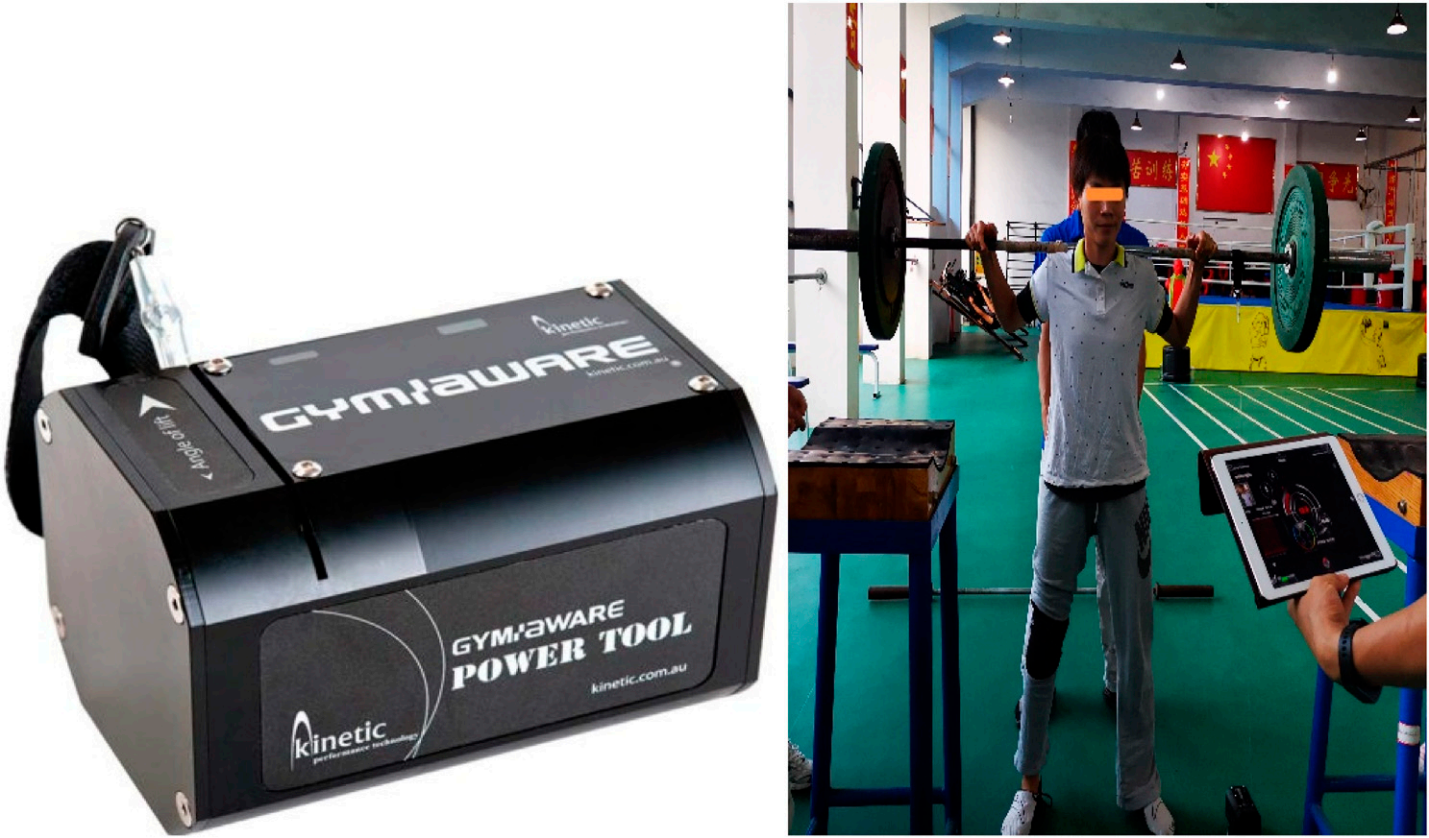
Schematic diagram of the GymAware Power Testing System.

GymAware (Orange et al., 2018b) was utilized to predict the 1RM strength in squatting and bench press exercises for athletes. The principle relies on the force-velocity curve and the relationship P=F *V, wherein the velocity generated by athletes in pushing the barbell was used for calculations (Figure 1). The force-velocity curve demonstrated that the predicted 1RM strength could be calculated when the velocity dropped below 0.3 m/s. The utilization of GymAware to measure 1RM presents advantages such as high accuracy, and prevents direct athlete-induced fatigue, mitigating injury risks during training. It is recommended to conduct five sets of 1RM prediction, commencing with 50% of the self-estimated maximum load and gradually progressing to 60%–70%, and 75%–80%, with repetitions of 3-3-2-2-1 for each set, with 2–3 min of rest in between. The goal is to lift as quickly as possible. For bench press and squat 1RM predictions, at least three different results under varying loads need to be recorded using the GymAware software. Prior to the maximal testing, standard dynamic warm-up exercises were executed. The initial weight was set at 50% of the self-estimated 1RM, wherein the participant performed five repetitions at this weight. Subsequently, 60% and 70% of the estimated 1RM were used for three and two repetitions, respectively. Following warm-up, participants rested for 3 min, performed one repetition at 80% of the estimated 1RM, and then another repetition at 90% of the estimated 1RM. At this point, participants attempted to reach the maximum possible load (Helms et al., 2016). At least a 3-min and a maximum of 5-min rest interval was maintained between sets.

Tables 1, 2 presents the statistical summary of 1RM bench press and squat weights for the experimental groups. The 1RM bench press weights for the UNI, UNI + BI, and BI groups were 46.14 ± 12.21 kg, 50.68 ± 11.59 kg, and 46.27 ± 11.71 kg, respectively (*F* = 0.5390, *p* > 0.05); similarly, the 1RM squat weights were 72.53 ± 15.19 kg, 72.71 ± 20.02 kg, 68.93 ± 20.78 kg, respectively (*F* = 0.1515, *p* > 0.05).

**TABLE 1:** UNI, UNI + BI, and BI group basic information (*n* = 30).

	(UNI, *n* = 10)	(UNI + BI, *n* = 10)	(BI, n = 10)	*F*	*p*
Height (m)	1.73 ± 0.08	1.7 ± 0.06	1.74 ± 0.06	0.6205	0.544
Weight (Kg)	55.42 ± 5.85	54.73 ± 5.33	59.67 ± 8.39	1.874	0.170
BMI (kg/m^2^)	19.09 ± 1.58	18.94 ± 1.49	19.56 ± 2.81	1.443	0.251

BMI, body mass index.

**TABLE 2:** Bench press and deep squat 1RM predicted data (kg).

	UNI group	UNI + BI group	BI group	*F*	*p*
Bench press 1RM (kg)	46.14 ± 12.21	50.68 ± 11.59	46.27 ± 11.71	0.5390	0.5885
Squat 1RM (kg)	72.53 ± 15.19	72.71 ± 20.02	68.93 ± 20.78	0.1515	0.8600

### 2.4 Protocol

The training protocol for this study was primarily based on the exercise design proposed by Carter and Greenwood (2014) for complex resistance training. Standard strength training programs for athletes often incorporate a combination of closed-chain and open-chain exercises to enhance upper and lower extremity strength, sport-specific performance, and injury prevention (Prokopy et al., 2008). Open-chain exercises allow endpoints to move freely with or without external resistance, such as in boxing, throwing, kicking, or using free weights for training (West et al., 2013).

Training was divided into two phases. The foundation training phase (first 2 weeks) was implemented to adapt athletes, especially beginners, to the demands of the exercises, and was performed at a slower pace. This approach aimed to prevent inexperienced trainees from performing overly vigorous movements that might compromise balance and lead to injuries. During the implementation of the complex resistance training program, attention was given to correcting posture and ensuring correct form development. A load of approximately 75%–80% of the specified resistance was used during this phase, emphasizing exercise stability rather than speed. The improvement training phase (last 6 weeks) included complex resistance training at a load of 80%–85%. Athletes were required to possess proficient technique and execute the exercises rapidly. Training sessions were divided into warm-up, implementation, and cool-down stretching sections.

The intervention was overseen and implemented by two coaches. Equal distribution of training volume was ensured (3 sets of resistance * 5 repetitions +15–30 rapid plyometric movements), with 3 min of active rest intervals (Table 3). In the UNI group, the resistance was not <50% of the bilateral 1RM, and in the bilateral group, it was not <80–85% of 1RM. The training protocol followed the principles of targeted resistance training design. For the UNI + BI group, the training sessions alternated between UNI and BI exercises. The training sequence consisted of upper body (single and double) exercises followed by lower body (double and single-sided) exercises. In this group, the first session involved single-sided training, and the second session was bilateral training, alternating 12 times each. The UNI and BI groups underwent 24 sessions of complex resistance training over 8 weeks (Bird et al., 2005).

**TABLE 3:** Training protocol.

Category		Stimulation type	Rapid stretch-shortening cycle training + ballistic training
Unilateral training	Upper extremity	Single-arm landmine barbell Press (50% 1RM, 3 sets of 5 repetitions) or single-arm dumbbell bench press (80%–50% 1RM, 3 sets of 5 repetitions)	One-hand box push-up (3 sets of 10 repetitions) followed by one-hand seated medicine ball throw
	Lower extremity	Bulgarian split squat, kettlebell heel-raised split squat (50% 1RM, 1RM, 3 sets of 5 repetitions)	Split jump (3 sets of 15–30 repetitions)
		Bulgarian unilateral heel raise (50% 1RM, 1RM, 3 sets of 5 repetitions)	Single-leg hurdle jump (0.25 m) (3 sets of 15–30 repetitions)
Bilateral training	Upper extremity	Bilateral bench press (80%–85% 1RM, 3 sets of 5 repetitions)	Clap push-ups (3 sets of 10 repetitions) followed by seated medicine ball throw (3 sets of 15–30 repetitions)
	Lower extremity	Bilateral squat (80%–85% 1RM, 3 sets of 5 repetitions)	Box jumps (40 cm) (3 sets of 15–30 repetitions)
		Heel raise exercise (80%–85% 1RM, 3 sets of 5 repetitions)	Hurdle jump (3 sets of 15–30 repetitions)"

### 2.5 Statistical analyses

We employed a between-group design with different intervention strategies as contrasts. All data from the various intervention training trials (UNI, UNI + BI, and BI, totaling three groups) were recorded and stored using Excel. The collected experimental data were subjected to statistical analysis using SPSS version 26.0. Prior to conducting the statistical analyses, the normal distribution of each group’s data was verified through the Passed Normality Test (*p* > 0.05) and homogeneity of variance was assessed using the Brown–Forsythe test (*p* > 0.05). Paired sample t-tests were applied to assess within-group differences between pre-test and post-test data, whereas a one-way analysis of variance (ANOVA) was used for between-group comparisons. Cohen’s d = M_diff/SD_pre, where M_diff represents the mean difference and SD_ pre represents the standard deviation of the pre -test.GraphPad Prism 6 software was utilized to generate linear graphs and bar charts as necessary.

## 3 Results

### 3.1 Within-group comparisons of different resistance exercise performances

#### 3.1.1 Pre- and post-performance in for the UNI group

##### 3.1.1.1 Bench press

**FIGURE 2:**
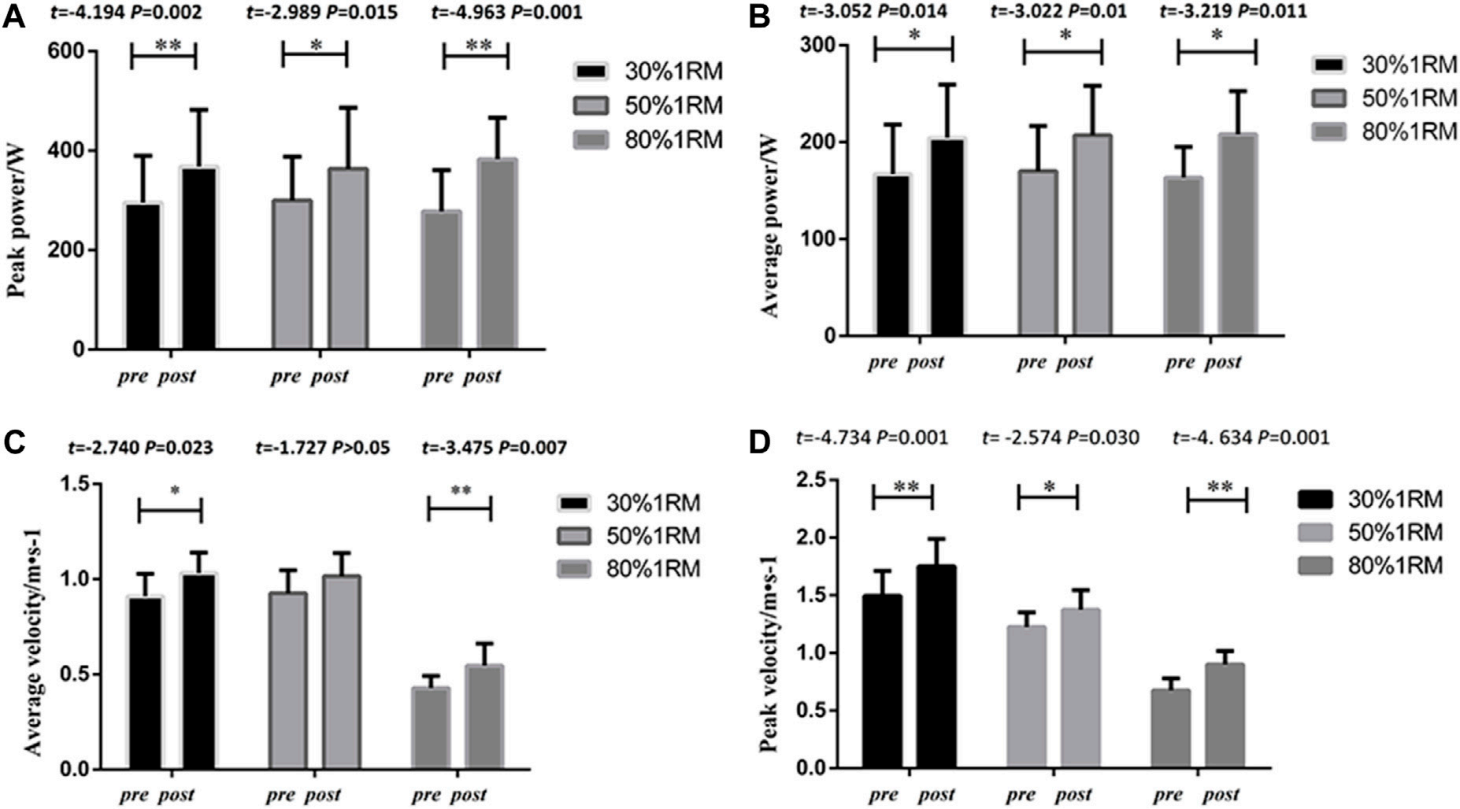
Pre- and post-intervention performance in different proportional 1RM bench press tests for the UNI Group.

The results of upper body bench press performance in the UNI group are presented in Figure 2. Following 8 weeks of intervention training, significant differences were observed in peak power, average power, peak velocity, and average velocity indicators at different loads (30%, 50%, and 80% of 1RM) for bench press in the UNI group, when comparing pre-test and post-test data. Significant differences were found in peak power indicators for 30% 1RM (*p* < 0.01,*Cohen’s d = 0.76*,*95%CI* = 33.57–112.17), 50% 1RM (*p* < 0.05,*Cohen’s d = 0.62,95%CI* = 8.63–103.36), and 80% 1RM (*p* < 0.01,*Cohen’s d = 1.26,95%CI* = 57.30–153.32). Statistical analysis of average power indicators revealed significant differences between 30% 1RM (*p* < 0.05,*Cohen’s d = 0.72,95%CI* = 9.64–64.90), 50% 1RM (*p* < 0.01,*Cohen’s d = 0.81,95%CI* = 9.36–58.67), and 80% 1RM (*p* < 0.05,*Cohen’s d = 1.01,95%CI* = 13.30–76.21). Significant differences were also observed in peak velocity indicators for 30% 1RM (*p* < 0.01,*Cohen’s d = 1.18,95%CI* = 0.134–0.379), 50% 1RM (*p* < 0.05,*Cohen’s d = 1.07,95%CI* = 0.0179–0.278), and 80% 1RM (*p* < 0.01,*Cohen’s d = 1.46,95%CI* = 0.115–0.336). The statistical analysis of average velocity indicators indicated significant changes in 30% 1RM (*p* < 0.05,*Cohen’s d = 1.0,95%CI* = 0.021–0.224), 50% 1RM (*p* > 0.05), and 80% 1RM (*p* < 0.01,*Cohen’s d = 1.83,95%CI* = 0.040–0.193).

#### 3.1.1.2 Lower limb squat indicators in the UNI group

**FIGURE 3:**
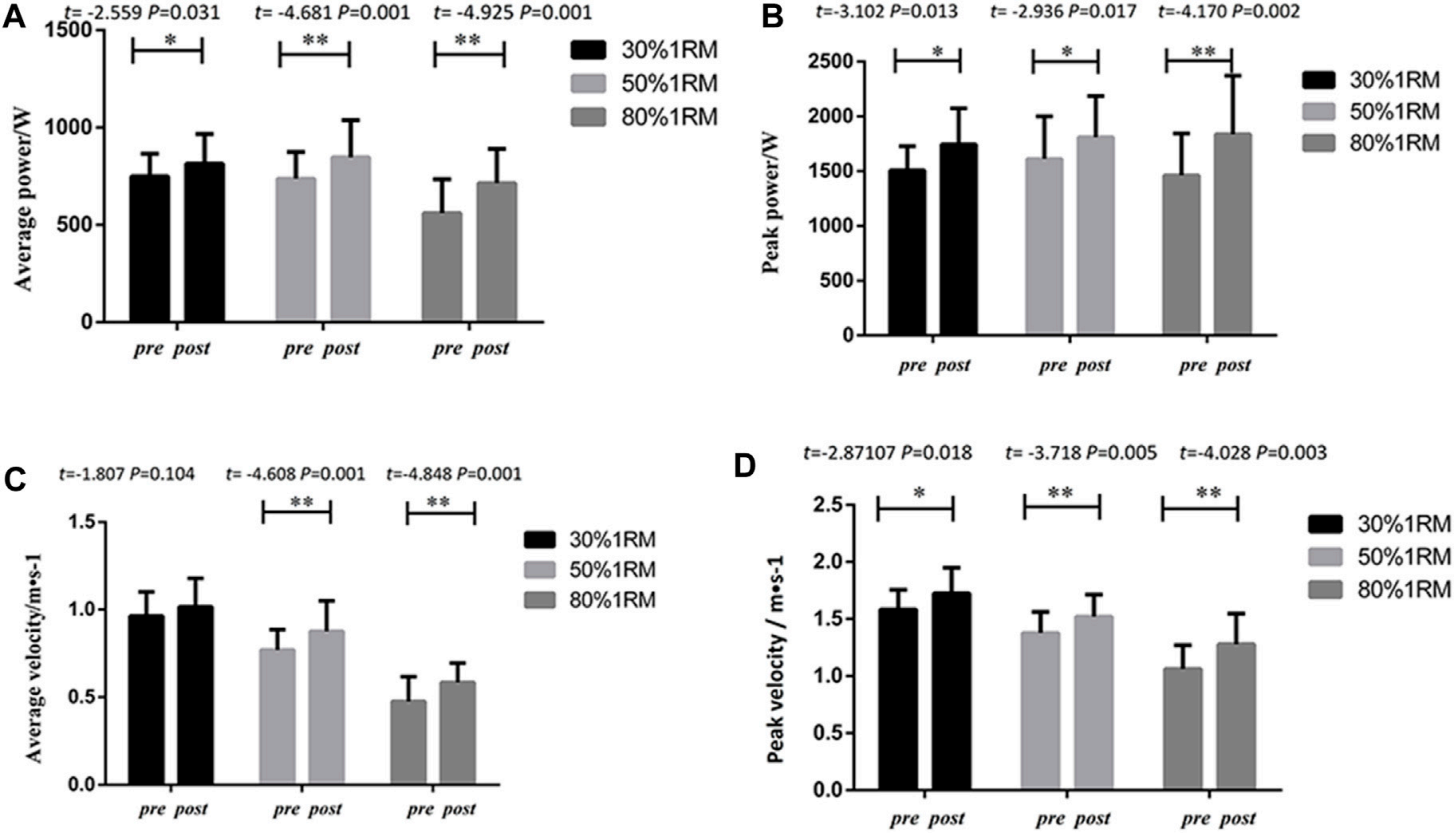
Pre- and post-intervention performance in different proportional 1RM squat tests for the UNI Group.

As shown in Figure 3, following 8 weeks of intervention training, significant differences were observed in peak power, average power, peak velocity, and average velocity indicators at different loads (30%, 50%, and 80%1RM) for squat exercise in the UNI group, when comparing pre-test and post-test data. Significant differences were found in peak power indicators for 30% 1RM (*p* < 0.05, *Cohen’s d = 1.08,95%CI* = 64.22–410.12), 50% 1RM (*p* < 0.05*,Cohen’s d = 0.76,95%CI* = 20.976–287.82), and 80% 1RM (*p* < 0.01,*Cohen’s d = 0.97,95%CI* = 172.14–580.34). Statistical analysis of average power indicators revealed significant differences in 30% 1RM (*p* < 0.05, *Cohen’s d = 0.55, 95%CI* = 7.58–123.03), 50% 1RM (*p* < 0.01,*Cohen’s d = 0.80,95%CI* = 56.84–163.16), and 80% 1RM (*p* < 0.05,*Cohen’s d = 0.88,95%CI* = 83.34–224.94). Significant differences were also observed in peak velocity indicators at 30% 1RM (*p* < 0.05, *Cohen’s d = 0.82,95%CI* = 0.030–0.253), 50% 1RM (*p* < 0.05,*Cohen’s d = 0.73,95%CI* = 0.056–0.233), and 80% 1RM (*p* < 0.01, *Cohen’s d = 1.04,95%CI* = 0.095–0.338). The statistical analysis of average velocity indicators indicated significant changes in 30% 1RM (*p* > 0.05), 50% 1RM (*p* < 0.01,*Cohen’s d = 0.91,95%CI* = 0.053–0.157), and 80% 1RM (*p* < 0.01,*Cohen’s d = 0.71,95%CI* = 0.058–0.159).

### 3.1.2 Pre- and post-performance in 1RM for the UNI + BI group

#### 3.1.2.1 Bench press

**FIGURE 4:**
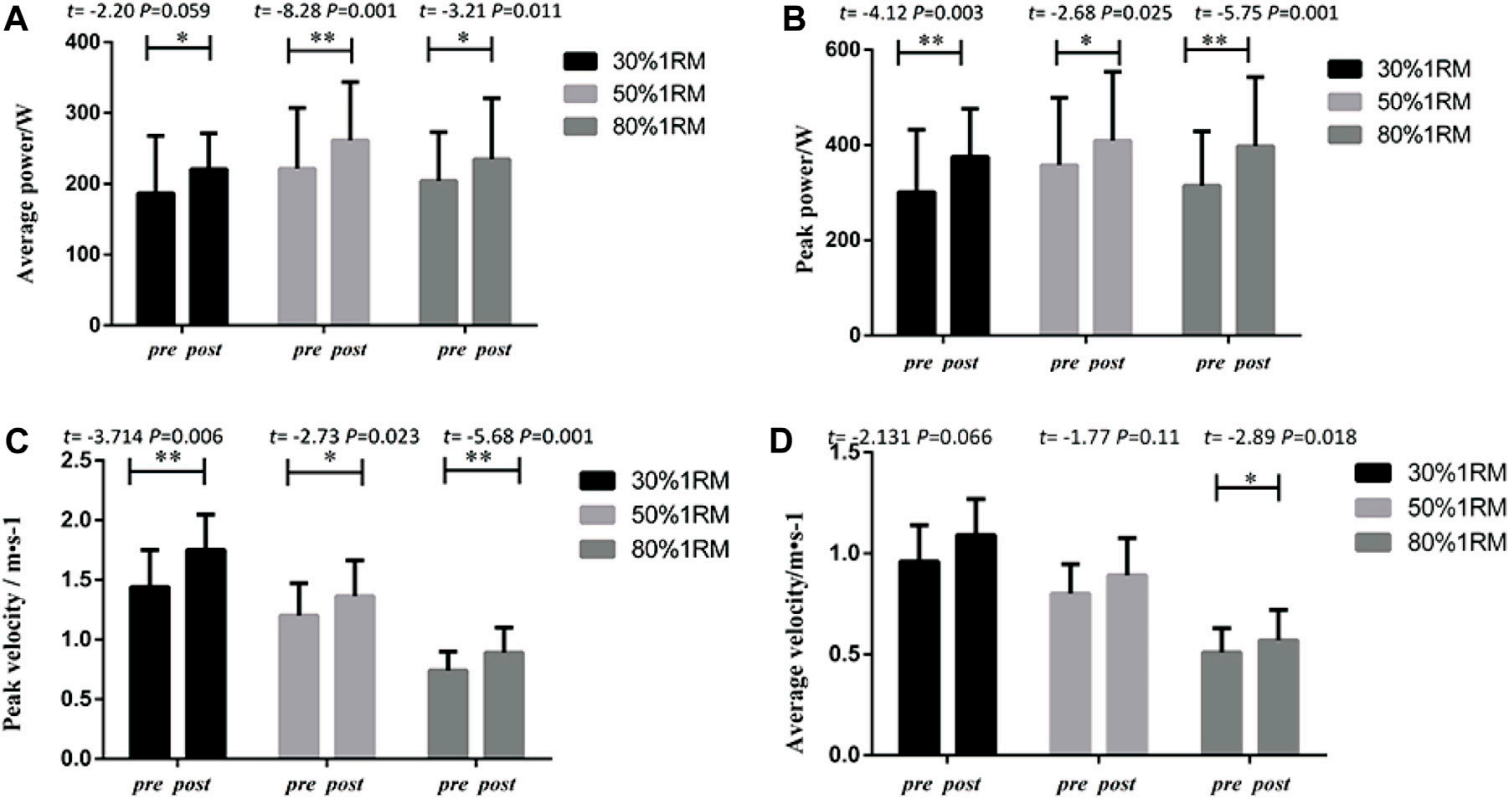
Pre- and post-intervention performance in different proportional 1RM bench press tests for the UNI + BI Group.

As shown in Figure 4, following 8 weeks of intervention training, significant differences were observed in peak power, average power, peak velocity, and average velocity indicators at different loads (30%, 50%, and 80% 1RM) for bench press exercises in the UNI + BI group, when comparing pre-test and post-test data. Significant differences were found in peak power indicators at 30% 1RM (*p* < 0.01, *Cohen’s d = 0.62,95% CI* = 45.79–105.12), 50% 1RM(*p*<0.01, *Cohen’s d = 0.57,95%CI* = 55.784–98.33), and 80% 1RM (*p* < 0.01*,Cohen’s d = 0.54,95%CI* = 45.79–105.11). Statistical analysis of average power indicators revealed significant differences in 30% 1RM (*p* < 0.05, *Cohen’s d = 0.83,95%CI* = 32.35–65.08), 50% 1RM (*p* < 0.01, *Cohen’s d = 0.55,95%CI* = 29.88–60.29) and 80% 1RM (*p* < 0.05, *Cohen’s d = 0.37,95%CI* = 6.41–47.03). Significant differences were also observed in peak velocity indicators at 30% 1RM (*p* < 0.01, *Cohen’s d = 1.14,95%CI* = 0.160–0.475), 50% 1RM (*p* < 0.05, *Cohen’s d = 0.66,95%CI* = 0.0526–0.300), and 80% 1RM (*p* < 0.01, *Cohen’s d = 0.70,95%CI* = 0.042–0.197). The statistical analysis of average velocity indicators indicated the following results: 30% 1RM (*p* > 0.05); 50% 1RM (*p* > 0.05); and 80% 1RM (*p* < 0.05*,Cohen’s d = 0.82,95%CI =* 0.0102–0.103). Except for the average velocity indicators, the UNI + BI group exhibited significant and highly significant differences in peak power, average power, peak velocity, and average velocity indicators between pre-test and post-test analyses.

#### 3.1.2.2 Lower limb squats

**FIGURE 5:**
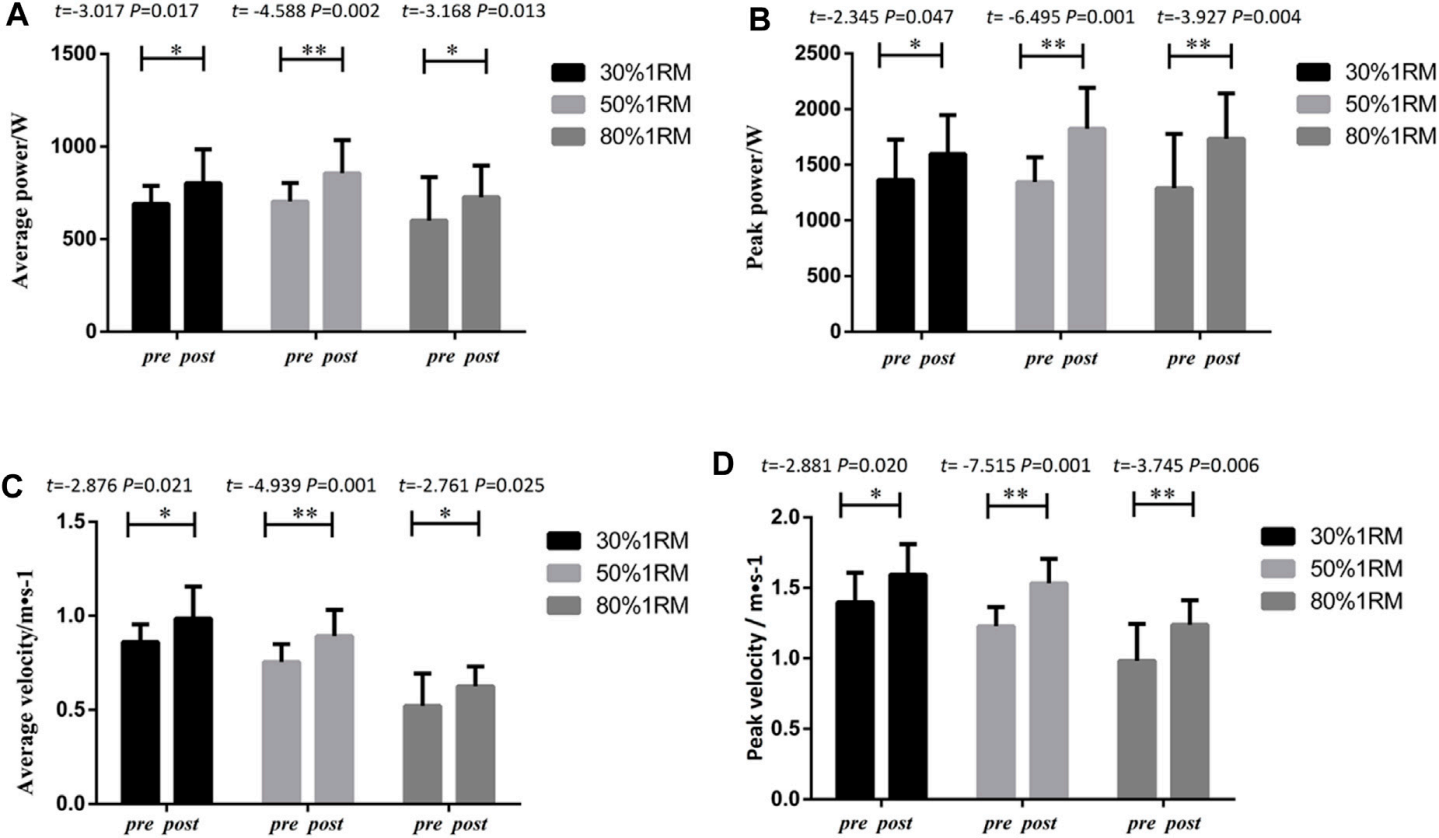
Pre- and post-intervention performance in different proportional 1RM squat tests for the UNI + BI Group.

As illustrated in Figure 5, following 8 weeks of intervention training, significant differences were observed in peak power, average power, peak velocity, and average velocity indicators for squats at different loads (30%, 50%, and 80% 1RM) in the UNI + BI group, when comparing pre-test and post-test data. Significant differences were found in peak power indicators at 30% 1RM (*p* < 0.05, *Cohen’s d = 0.65,95%CI* = 23.75–427.60), 50% 1RM (*p* < 0.01,*Cohen’s d = 1.78,95%CI* = 225.49–561.04), and 80% 1RM (*p* < 0.01,*Cohen’s d = 0.80,95%CI* = 82.74–397.71). Statistical analysis of average power indicators revealed significant differences in 30% 1RM (*p* < 0.05,*Cohen’s d = 1.04,95%CI* = 33.91–184.43), 50% 1RM (*p* < 0.01,*Cohen’s d = 1.25,95%CI* = 66.85–213.97), and 80% 1RM (*p* < 0.05,*Cohen’s d = 0.50,95%CI* = 28.08–199.11). Significant differences were also observed in peak velocity indicators at 30% 1RM (*p* < 0.05, *Cohen’s d =* 0.95,*95%CI = 0.047 to 0.327*), 50% 1RM (*p* < 0.01,*Cohen’s d = 1.44,95%CI = 0.176 to 0.380*), and 80% 1RM (*p* < 0.01, *Cohen’s d =* 0.56,*95%CI = 0.079 to 0.377*). The statistical analysis of average velocity indicators indicated the following results: 30% 1RM (*p* < 0.05,*Cohen’s d = 1.33,95%CI* = 0.0327–0.205); 50% 1RM (*p* < 0.01,*Cohen’s d = 1.44,95%CI = 0.064 to 0.188*) and 80% 1RM (*p* < 0.05,*Cohen’s d = 0.56,95%CI* = *0.013 to 0.174*).

### 3.1.3 Pre- and post-performance for the BI group

#### 3.1.3.1 Bench press

**FIGURE 6:**
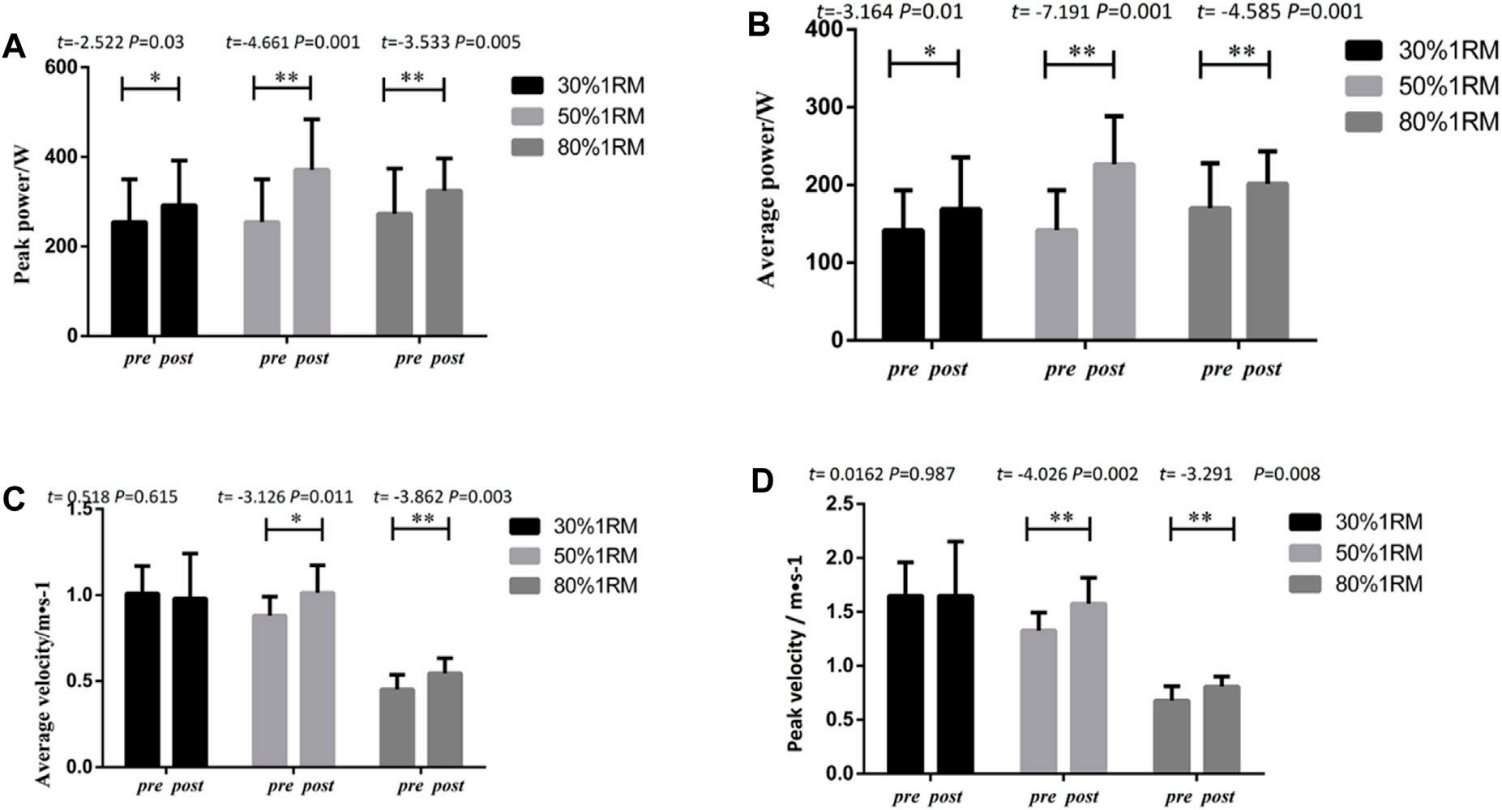
Pre- and post-intervention performance in different proportional 1RM bench press tests for the BI Group.

As presented in Figure 6, the BI group exhibited significant differences in peak power, average power, peak velocity, and average velocity indicators for bench press exercises at different loads (30%, 50%, and 80% 1RM) following 8 weeks of intervention training, when comparing pre-test and post-test data. Significant differences were observed in peak power indicators (30% 1RM, *p* < 0.05, *Cohen’s d = 0.51,95%CI =*7.48 to 28.97; 50% 1RM, *p* < 0.01, *Cohen’s d = 0.70,95%CI =*2.073 to 70.27% and 80% 1RM, *p* < 0.01, *Cohen’s d = 0.73,95%CI =*21.14–49.19), average power indicators (30% 1RM *p* < 0.01, *Cohen’s d = 0.40,*95%CI = 3.91 to 77.43; 50% 1RM *p* < 0.01, *Cohen’s d = 0.88,95%CI =* 14.30 to 138.73; 80% 1RM *p* < 0.01, *Cohen’s d = 0.81,95%CI =*38.16–87.46) and peak velocity indicators (30% 1RM, *p* > 0.05; 50% 1RM, *p* < 0.01, *Cohen’s d = 1.68,*95%CI = 0.219 to 0.633; 80% 1RM, *p* < 0.01,*Cohen’s d = 1.45,*95%CI = 0.079–0.230). Similarly, significant differences were found in average velocity indicators for 30% 1RM (*p* > 0.05), 50% 1RM (*p* < 0.05, *Cohen’s d = 1.97,* 95%CI = 0.159–0.367) and 80% 1RM (*p* < 0.01, *Cohen’s d = 1.83,*95%CI = 0.079–0.230).

#### 3.1.3.2 Lower limb squats

**FIGURE 7:**
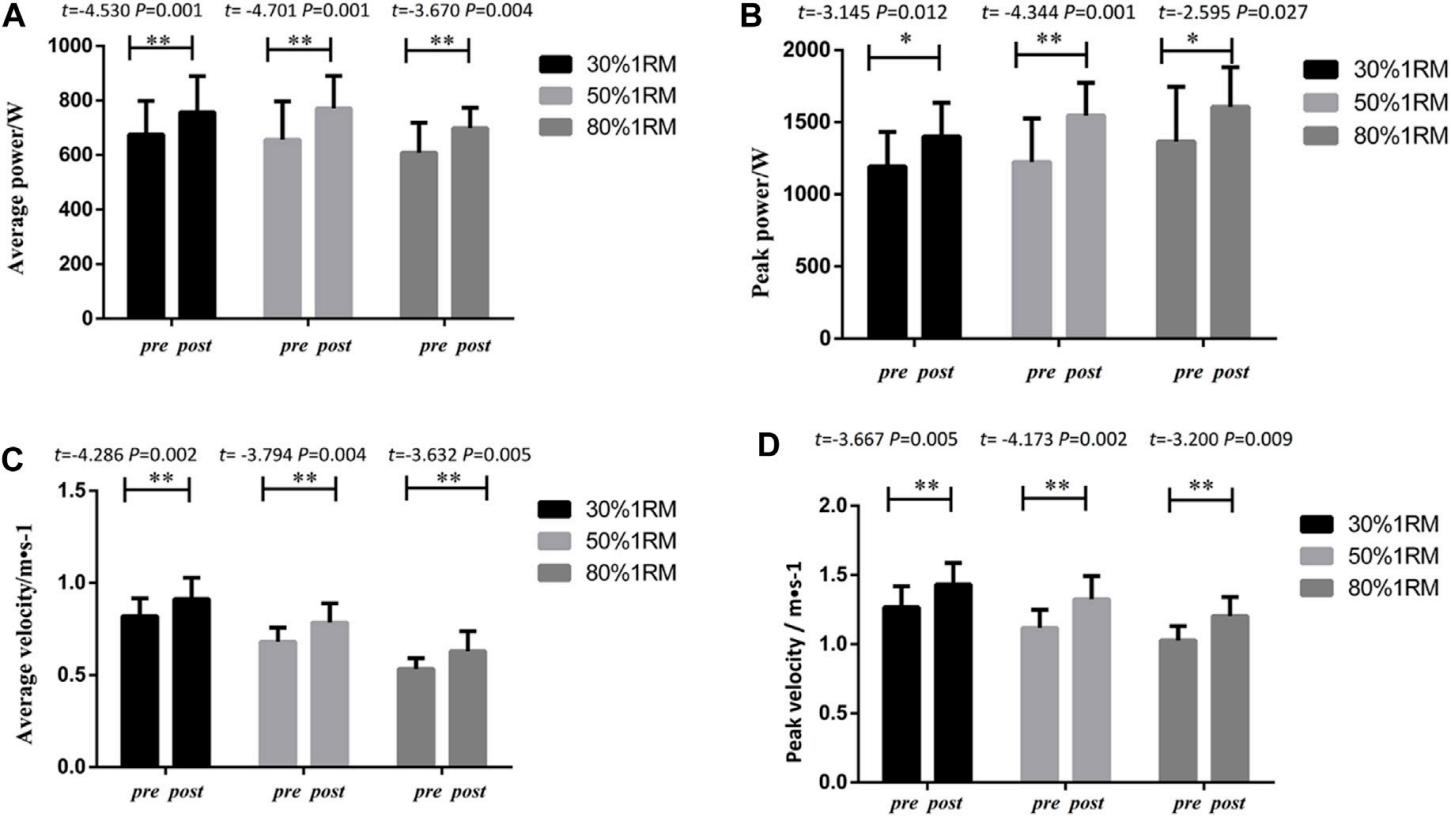
Pre- and post-intervention performance in different proportional 1RM squat tests for the BI Group.

As shown in Figure 7, the BI group demonstrated significant differences in peak power, average power, peak velocity, and average velocity indicators for squat exercises at different loads (30%, 50%, and 80% 1RM) following 8 weeks of intervention training, when comparing pre-test and post-test data. Significant differences were observed in peak power indicators 30% 1RM(*p*<0.05, *Cohen’s d = 0.98,95%CI =*43.615–383.15) 50% 1RM(*p*<0.01, *Cohen’s d = 1.33,95%CI =*176.05–521.89) and 80% 1RM(*p*<0.05, *Cohen’s d = 0.67,95%CI =*176.05–521.89), average power indicators30% 1RM(*p*<0.01, *Cohen’s d = 0.70,95%CI =*34.43–126.54) 50% 1RM(*p*<0.01, *Cohen’s d = 1.01,95%CI =*66.40–180.44) and 80% 1RM(*p*<0.01, *Cohen’s d = 0.95,95%CI =*41.87–155.74), and peak velocity indicators (30% 1RM, *p* < 0.01, *Cohen’s d = 1.13,95%CI =*0.057 to 0.287; 50% 1RM, *p* < 0.01,*Cohen’s d = 1.83,95%CI =*0.107 to 0.340; 80% 1RM, *p* < 0.01, *Cohen’s d = 1.72,95%CI =*0.062–0.319). Similarly, significant differences were found in average velocity indicators (30% 1RM, *p* < 0.01, *Cohen’s d = 0.90,95%CI =*0.0374 to 0.149; 50% 1RM, *p* < 0.01, *Cohen’s d = 1.57,95%CI =*0.046 to 0.177; 80% 1RM, *p* < 0.05, *Cohen’s d = 1.83,95%CI =*0.044–0.167).

### 3.2 Intergroup comparison of 1RM ratio resistance exercise performances

#### 3.2.1 Indicators at 30% 1RM

The overview of the growth values of straight punch indicators for upper limb evaluation before and after the intervention is presented in Table 4. The peak power indicators for the UNI, UNI + BI, and BI groups increased by 79.68 ± 45.86 W, 81.97 ± 34.57 W, and 39.24 ± 21.26 W, respectively. The average power indicators increased by 40.72 ± 34.54 W, 48.72 ± 22.88 W, and 19.25 ± 13.53 W for the three groups, respectively. The peak velocity indicators increased by 0.27 ± 0.15 m/s, 0.32 ± 0.22 m/s, and 0.27 ± 0.27 m/s, respectively, while the average velocity indicators increased by 0.15 ± 0.11 m/s, 0.17 ± 0.13 m/s, and 0.12 ± 0.14 m/s for the UNI, UNI + BI, and BI groups, respectively. Significant differences were observed in peak power and average power indicators for upper limbs at 30% 1RM (F = 4.622, F = 3.468, *p* < 0.05). LSD *post hoc* comparisons showed significant differences between the UNI and BI groups (UNI vs BI, *p* < 0.05, 95% CI = 9.429–85.82), as well as between the UNI + BI and BI groups (UNI + BI vs BI, *p* < 0.05, 95% CI = 5.550–79.91) in terms of peak power and between UNI + BI and BI groups (UNI + BI vs BI, *p* < 0.05, 95% CI = 2.463–56.47) in terms of average power. However, no statistically significant differences were found in peak velocity and average velocity (*p* > 0.05). These results suggest that, after 8 weeks of compound training, significant differences exist among groups in terms of peak power and average power indicators for the upper limbs at 30% 1RM, with UNI and UNI + BI groups surpassing the BI group.

**TABLE 4:** Output power for upper and lower extremities before and after 30% 1RM intervention.

	Indicators	UNI difference	UNI + BI difference	BI difference	*F*
Upper extremity	Peak power	79.68 ± 45.86	81.97 ± 34.57	39.24 ± 21.26	4.622*
	Average power	40.72 ± 34.54	48.72 ± 22.88	19.25 ± 13.53	3.666*
	Peak velocity	0.27 ± 0.15	0.32 ± 0.22	0.27 ± 0.27	0.488
	Average velocity	0.15 ± 0.11	0.17 ± 0.13	0.12 ± 0.14	0.146
Lower extremity	Peak power	295.85 ± 166.08	305.82 ± 194.2	148.78 ± 109.49	3.468*
	Average power	119.45 ± 30.09	129.09 ± 82.5	87.01 ± 48.59	1.857
	Peak velocity	0.18 ± 0.1	0.22 ± 0.16	0.18 ± 0.14	0.253
	Average velocity	0.08 ± 0.07	0.14 ± 0.09	0.1 ± 0.06	1.595

Regarding lower limb squat load assessment indicators, the peak power indicators increased by 295.85 ± 166.08 W, 305.82 ± 194.2 W, and 148.78 ± 109.49 W for the UNI, UNI + BI, and BI groups, respectively; average power indicators, by 119.45 ± 30.09 W, 129.09 ± 82.5 W, and 87.01 ± 48.59 W, respectively; peak velocity indicators, by 0.18 ± 0.1 m/s, 0.22 ± 0.16 m/s, and 0.18 ± 0.14 m/s, respectively; and average velocity indicators by 0.08 ± 0.07 m/s, 0.14 ± 0.09 m/s, and 0.1 ± 0.06 m/s, respectively. Significant differences were observed in peak power indicators for lower limb squat exercises at 30% 1RM among the three groups. Post hoc multiple comparisons revealed significant differences between the UNI and control groups (158.6 difference in means) and between the UNI + BI and control groups (168.5 difference in means) in terms of peak power. However, no significant intergroup differences were found in various aspects of strength output among the UNI and UNI + BI groups under different load conditions. These findings indicate that, after 8 weeks of compound training, significant differences only exist in terms of peak power indicators for lower limb squat exercises at 30% 1RM, with both UNI and UNI + BI groups surpassing the BI group.

#### 3.2.2 Indicators at 50% 1RM

The summary of upper limb strength indicators at 50% 1RM for the UNI, UNI + BI, and BI groups is provided in Table 5. The peak power indicators increased by 82.11 ± 40.01 W, 80.76 ± 28.66 W, and 77.92 ± 43.07 W for the UNI, UNI + BI, and BI groups, respectively; average power indicators, by 40.18 ± 30.13 W, 45.09 ± 22.64 W, and 40.09 ± 23.92 W, respectively; peak velocity indicators, by 0.22 ± 0.11 m/s, 0.26 ± 0.13 m/s, and 0.26 ± 0.13 m/s, respectively; and average velocity indicators by 0.11 ± 0.08 m/s, 0.15 ± 0.09 m/s, and 0.19 ± 0.1 m/s, respectively. Overall, there were no statistically significant intergroup differences in peak power, average power, peak velocity, and average velocity indicators among the three groups in the local training of 50% 1RM strength assessment.

**TABLE 5:** Overview of pre- and post-intervention differences in output power for upper and lower extremities at 50% 1RM.

	Indicators	UNI group difference	UNI + BI group difference	BI group difference	*F*
Upper extremity	Peak power	82.11 ± 40.01	80.76 ± 28.66	77.92 ± 43.07	0.029
	Average power	40.18 ± 30.13	45.09 ± 22.64	40.09 ± 23.92	0.1284
	Peak velocity	0.22 ± 0.11	0.22 ± 0.14	0.26 ± 0.13	0.2876
	Average velocity	0.11 ± 0.08	0.15 ± 0.09	0.19 ± 0.1	1.438
Lower extremity	Peak power	316.9 ± 116.94	437.47 ± 248.76	373.07 ± 213.42	1.698
	Average power	127.84 ± 51.3	140.41 ± 102.83	131.32 ± 69.36	0.288
	Peak velocity	0.17 ± 0.09	0.28 ± 0.14	0.22 ± 0.16	1.755
	Average velocity	0.12 ± 0.04	0.13 ± 0.09	0.11 ± 0.09	0.095

Regarding lower limb indicators at 50% 1RM, the output power and velocity indicators for the three groups increased by 316.9 ± 116.94 W, 437.47 ± 248.76 W, and 373.07 ± 213.42 W, respectively; average power indicators, by 127.84 ± 51.3 W, 140.41 ± 102.83 W, and 131.32 ± 69.36 W, respectively; peak velocity indicators, by 0.17 ± 0.09 m/s, 0.28 ± 0.14 m/s, and 0.22 ± 0.16 m/s, respectively; and average velocity indicators, by 0.12 ± 0.04 m/s, 0.13 ± 0.09 m/s, and 0.11 ± 0.09 m/s for the UNI, UNI + BI, and BI groups, respectively. The results indicate that there were no significant differences among the lower limb groups at 50% 1RM, suggesting that the 8-week intervention training did not lead to significant intergroup differences in the strength quality of both upper and lower limbs.

#### 3.2.3 Indicators at 80% 1RM

As shown in Table 6, in the bench press assessment, the UNI, UNI + BI, and BI groups exhibited increases in peak power indicators by 105.32 ± 67.11 W, 85.35 ± 37.58 W, and 62.82 ± 34.46 W, respectively. The average power indicators increased by 46.18 ± 42.31 W, 38.5 ± 22.54 W, and 35.17 ± 19.61 W for the three groups, respectively; peak velocity indicators, by 0.23 ± 0.15 m/s, 0.16 ± 0.07 m/s, and 0.15 ± 0.11 m/s, respectively; and average velocity indicators, by 0.12 ± 0.1 m/s, 0.10 ± 0.04 m/s, and 0.1 ± 0.08 m/s for the UNI, UNI + BI, and BI groups, respectively. Statistically, no significant intergroup differences were observed in peak power, average power, peak velocity, and average velocity indicators at 80% 1RM. These findings suggest that, after an 8-week intervention training, there were no significant differences in absolute strength quality at the upper limb level among the UNI, UNI + BI, and BI groups.

**TABLE 6:** Pre- and post-intervention differences in output power for upper and lower extremities at 80% 1RM

	Indicators	UNI difference	UNI + BI difference	BI difference	*F*
Upper extremity	Peak power	105.32 ± 67.11	85.35 ± 37.58	62.82 ± 34.46	1.909
	Average power	46.18 ± 42.31	38.5 ± 22.54	35.17 ± 19.61	2.641
	Peak velocity	0.23 ± 0.15	0.16 ± 0.07	0.15 ± 0.11	1.137
	Average velocity	0.12 ± 0.1	0.10 ± 0.04	0.1 ± 0.08	0.187
Lower extremity	Peak power	418.84 ± 251.35	415.61 ± 167.98	370.88 ± 186.38	0.179
	Average power	163 ± 81.71	152.12 ± 69.35	115.77 ± 58.53	1.231
	Peak velocity	0.22 ± 0.17	0.23 ± 0.21	0.21 ± 0.16	0.044
	Average velocity	0.12 ± 0.05	0.12 ± 0.08	0.11 ± 0.08	0.078

For the lower limb indicators, the UNI, UNI + BI, and BI groups exhibited increases in peak power indicators by 418.84 ± 251.35 W, 415.61 ± 167.98 W, and 370.88 ± 186.38 W, respectively. The average power indicators increased by 163 ± 81.71 W, 152.12 ± 69.35 W, and 115.77 ± 58.53 W for the three groups, respectively. The peak velocity indicators increased by 0.22 ± 0.17 m/s, 0.23 ± 0.21 m/s, and 0.21 ± 0.16 m/s, while the average velocity indicators increased by 0.12 ± 0.05 m/s, 0.12 ± 0.08 m/s, and 0.11 ± 0.08 m/s for the UNI, UNI + BI, and BI groups, respectively. Statistically, no significant intergroup differences were observed in peak power, average power, peak velocity, and average velocity indicators at 80% 1RM for the lower limb. Overall, these results indicate that the 8-week intervention training did not result in significant intergroup differences in the absolute strength quality indicators for both upper and lower limbs.

## 4 Discussion

### 4.1 Upper extremity strength exercise performance

Boxing is a sport that integrates both strength and speed, requiring athletes to demonstrate high-level performance in terms of quick punching speed and significant power (Liu et al., 2023). The localized training effects of bench press can rapidly translate into specialized techniques such as straight punches and jabs, effectively enhancing the punching speed and power of boxing athletes. López et al. (2019) found a significant positive correlation (*p* < 0.05) between the peak velocity of bench press at submaximal intensity and the peak velocity of the rear straight punch. Furthermore, the peak velocity of the front punch and bilateral bench press have no significant correlation under different load percentages of 1RM (*p* > 0.05) (López et al., 2019), and bench press training significantly improves power output in Olympic-level boxing athletes, enhancing punching effectiveness (Loturco et al., 2019). Therefore, the bench press technique is frequently utilized in many studies to assess the maximal strength, strength endurance, and explosive power of athletes’ upper body. (Jidovtseff et al., 2011; Pallares et al., 2016).

Following an 8-week intervention of complex resistance training, we observed significant improvements (*p* < 0.05, *p* < 0.01) in bench press performance at 30%, 50%, and 80% of 1RM for the UNI, UNI + BI, and BI groups, respectively. This implies that all three intervention methods positively impacted upper extremity explosiveness, strength endurance, and absolute strength, further confirming the capability of complex resistance training to enhance both absolute strength and explosive power. This observation aligns with the findings of Ebben et al. (2000) which suggested that bench press combined with medicine ball training constitutes a unique form of upper extremity complex resistance training (Ebben et al., 2000; Baker, 2003). Another study (Baker, 2003) focused on complex resistance bench press training, alternating between high-load and light-load training sessions, resulting in a significant increase in bench press power. However, inconsistent results were also reported, as highlighted by Farup and Sorensen (2010), who reported no significant difference in upper extremity activation before and after five sets of 1RM bench press tasks for eight male participants. The lack of significance was attributed to the limitations of a small sample size. Similarly, Cuevas-Aburto et al. (2020) reported outcomes from a 6-week assessment of different training strategies (high-load strength group and end-range release training group) for bench press; the high-load strength group significantly improved their 1RM bench press strength, while the peak velocity of bench press (20 kg) did not improve. Conversely, the low-load end-range release bench press training group effectively enhanced the peak velocity of bench press (20 kg) but did not enhance 1RM bench press strength. Therefore, we focused on complex resistance training primarily emphasizing end-range release training or rapid plyometric training for the targeted muscle, aimed at enhancing punching explosiveness. In comparing different intervention methods, only the UNI and UNI + BI groups significantly outperformed the BI group in the 30% 1RM bench press explosiveness test. However, the extent of improvement in strength endurance at 50% 1RM and maximum endurance strength at 80% 1RM remained consistent across the three groups.

### 4.2 Lower extremity resistance exercise performance

The punching power of boxers originates from the lower limbs (Turner et al., 2011; Liu et al., 2022). Filimonov et al. (1985) reported that, as athlete skill levels increase, the contribution of the lower limbs to punching power becomes more significant. (Filimonov et al., 1985). The vertical jump height (counter movement jump) of boxers was positively correlated with the total number of punches thrown in official matches and the height of the rear uppercut punch (*r* = 0.735 and *r* = 0.793) (Rimkus et al., 2019). Research has shown that compound resistance training significantly improves lower limb explosive strength (Poulos, 2018). Squat training enhances lower limb strength in boxers and improves punching effectiveness (Turner et al., 2011; McBride, 2010). Emily C Dunn reported that enhancing lower limb strength in boxers without increasing body weight positively affects punching effectiveness (Dunn et al., 2020). There is a considerable body of research on the impact of lower limb strength on punching effectiveness in boxing (Busko, 2019). Pierce et al. observed that to generate higher cumulative punching force, both the upper and lower limbs must effectively exert greater levels of muscular strength (Pierce et al., 2006). Athletes should focus on developing maximum strength in the lower limbs by using methods that induce positive neural adaptations (Loturco et al., 2015).

In our study, we observed improvements in various indicators of lower limb performance across all groups after 8 weeks of training (pre- and post-test comparisons). Paul. reported differences in lower limb kinematics and kinetics using different percentages of 1RM squat methods (Swinton et al., 2012). This indicates that different training strategies effectively enhance performance levels with different resistances within an 8-week timeframe. This finding aligns with previous literature reporting improvements in athlete performance through unilateral, unilateral plus bilateral, and bilateral lower limb rapid eccentric training (Ramírez-Campillo et al., 2015; Lee et al., 2018). However, when comparing between groups, significant differences were observed only in the peak power indicator at 30% 1RM (*p* < 0.05), with UNI and UNI + BI groups outperforming the BI group. During UNI and UNI + BI groups training, it is possible that unilateral training may elicit greater activation of deep muscle and core muscle groups compared to bilateral training. This enhanced activation may lead to improved performance at lighter loads, such as explosive strength levels.

Therefore, the main objective of this paper is to explore the differences among three specific training methods. UNI and UNI + BI groups compound resistance training methods are superior to bilateral compound resistance training in enhancing explosive strength at 30% 1RM.

## 5 Conclusion

After 8 weeks of combined resistance training, significant improvements were observed in the localized lower limb training effects for different strength tests among the boxers. In intergroup comparisons, the UNI and UNI + BI groups exhibited superior indicators of explosive strength at 30% 1RM compared to the bilateral group.

## Data Availability

The original contributions presented in the study are included in the article/Supplementary materials, further inquiries can be directed to the corresponding author.
